# Tendon Grafting

**Published:** 1889-06-08

**Authors:** 


### TENDON GRAFTING.

Everybody knows something about skin grafting; the knowledge of tendon grafting, and, indeed, tendon grafting itself, is by no means so common. At the Clinical Society of London Mr. Mayo Robson recently related the history of an interesting case of this operation. The forearm and hand of a patient had been severely injured. All the muscles of the back of the hand, and their tendons as well, had been torn off, and the palm was much lacerated. The effect of such an injury, if untreated, would have been that though the patient might close his hand he could not open it again. Mr. Robson therefore took a piece of tendon four and a half inches long, which was hanging from the front of the hand, and stitched it to the remains of the tendon at the back of the forefinger and the muscular structure coming from the arm, thus joining the finger by muscular and tendinous connection with the only source of living movement possible to it. The wound was treated antiseptically, no sloughing took place, and the stitched-in tendon united perfectly at both ends. The result was that not only was the patient's hand preserved, but the use of the finger was also restored. Not very many years ago the only treatment that would have been thought of in such a case would have been the removal of the whole hand, or at least some of the fingers, and the substitution of a "Captain Cuttle's hook."

